# Supplementary material for: WordCommentsAnalyzer: A windows software tool for qualitative research
Source: F1000Res. 2018 Sep 4;7:536. Originally published 2018 May 3. [Version 2] doi: 10.12688/f1000research.14819.2 (PMC6137411; doi:10.12688/f1000research.14819.2)
Supplement: Supplementary file 3 [file f1000research-7-17614-s0002.tgz › b2a79496-4ada-47c4-9682-a99ce867836d.zip!/Eight Iranian Health Journals/Yafteh _48.docx]

© Yafteh  . This work is licensed under CC BY 4.0 (https://creativecommons.org/licenses/by/4.0/).

Volume & Issue: دوره 9 - شماره 1

Title: بررسی آگاهی ساکنین شهر تهران از آلودگی های زیست محیطی و عوارض بهداشتی مربوطه و تعیین منابع اطلاعاتی آنان

http://yafte.lums.ac.ir/article-1-20-fa.html

مقدمه : مواجهه های محیطی معمولاً با عوامل شیمیائی، فیزیکی و میکروبیولوژیک رخ میدهد. سازمان جهانی بهداشت در گزارش سال 2002 خود بار منتسب به 25 عامل خطر را محاسبه نموده که 7 عامل از این عوامل محیطی تلقی میشود. با توجه به اینکه در خصوص موضوع آلودگی محیط زیست مطالعه ای جامع در تهران صورت نگرفته است این مطالعه به منظور بررسی آگاهی ساکنین شهر تهران از آلودگی های زیست محیطی و عوارض بهداشتی مربوطه و تعیین منابع اطلاعاتی آنان انجام شد. Ø مواد و روشها: مطالعه حاضر بصورت مقطعی بر روی 1596 نفر از ساکنین بالای 18 سال شهر تهران انجام شده است. نحوه نمونهگیری بصورت تصادفی ساده بود. داده ها به روش مصاحبه تلفنی جمع آوری گردید. برای تعیین ارتباط متغیرهای کیفی از آزمون کای دو و در صورت لزوم از شاخص OR و حدود اطمینان آن استفاده شد. روایی سؤالات در هر حیطه با استفاده از شاخص آلفای کرونباخ مورد بررسی قرار گرفت. پایایی ساختاری سؤالات در هر حیطه با استفاده از روش تجزیه تحلیل مولفه های اصلی بررسی گردید. Ø یافته ها: نتایج نشان داد در مجموع 2/54% از افراد مورد بررسی دارای آگاهی مناسب در زمینه آلودگیهای زیست محیطی و عوارض بهداشتی مربوطه بوده اند. آگاهی زنان، جوانان (گروه سنی 18 تا 20 سال)، افراد دارای تحصیلات دانشگاهی در سطح دکترا، ساکنین غرب و شمال شهر و کسانی که منبع کسب اطلاعات آنها در این مورد رادیو و تلویزیون بوده در این زمینه بیشتر بوده است. اختلاف آگاهی افراد مورد بررسی در مورد آلودگیهای زیست محیطی و عوارض بهداشتی مربوطه با تحصیلات و منبع کسب اطلاعات آنها از نظر آماری معنی دار بوده است. Ø بحث و نتیجه گیری: با توجه به یافته های این مطالعه گرچه آگاهی شهروندان تهرانی در مورد برخی از موضوعات زیست محیطی و نهادهایی که در زمینه کنترل آلودگیهای زیست محیطی در کشور فعالیت دارند پایین به نظر میرسد اما در مجموع آگاهی مردم در تهران در این زمینه به ویژه در مشکلات بهداشتی ناشی از آلودگی هوا مناسب ارزیابی می گردد. با توجه به اهمیت نقش عوامل زیست محیطی در سلامتی جوامع و افزایش روز افزون آنها, بدلیل گسترش زندگی ماشینی به ویژه در شهرهای بزرگ کشور لازم است جهت شناسائی و کنترل این عوامل پژوهشهای مختلفی انجام شود. انجام مطالعات مداخله ای جهت پیدا کردن راهکار مناسب و عملیاتی کردن آنها از اولویت بسیار بالایی در این زمینه برخوردار است.

Title: بررسی ارتباط سیکل قاعدگی با میزان مصرف مپریدین و متوکلوپرامید جهت کنترل درد و تهوع و استفراغ پس از اعمال جراحی زنان

http://yafte.lums.ac.ir/article-1-21-fa.html

مقدمه: درد و تهوع و استفراغ پس از عمل ( PONV ) از علل بزرگ ناخوشی پس از عمل و بیهوشی می باشند. فاکتور های بسیاری بر بروز آنها موثرند که یکی از آن فاکتور ها ممکن است سیکل قاعدگی باشد. در این تحقیق اثر فاز های مختلف سیکل قاعدگی بر میزان نیاز به داروی ضد درد و ضد استفراغ بعد از عمل جراحی مورد ارزیابی قرار گرفت. مواد و روشها: در این مطالعه مقطعی آینده نگر دو سویه کور از 63 بیمار کاندید عمل جراحی ترمیم سیستوسل- رکتوسل که شرایط لازم برای ورود به مطالعه را داشتند، پس از گرفتن رضایت نامه، تاریخچه سیکل قاعدگی آنها گرفته شد. اولین روز قاعد گی قبلی را روز اول سیکل شمرده و روز انجام عمل جراحی بر اساس آن حساب گردید و بیماران به 4 گروه تقسیم شدند 1- فاز قاعدگی و قبل از قاعدگی (روز 25 تا 26) 2- فاز فولیکولار (روز 8 تا 12) 3- فاز تخمک گذاری (روز 13 تا 15) 4- فاز لوتئال (روز 20 تا 24). تمام بیماران بصورت استاندارد تحت بیهوشی عمومی قرار گرفتند. پس از اتمام عمل در صورتیکه بیماران در ریکاوری درجه دردی بیش از 5 برطبق VAS ( visual Analogue Scale ) داشتند، مپریدین ( mg/kg 1) وریدی دریافت کرده و سپس به بخش منتقل میشدند. در بخش در صورت وجود درد با درجه بیش از 5 بر طبق VAS مپریدین ( mg/kg 1) عضلانی و در صورت استفراغ، متوکلوپرامید ( mg/kg 3/0) وریدی آهسته برای بیماران تجویز می شد. طی 24 ساعت اول بعد از عمل درجه تهوع و استفراغ و درجه درد و نیز میزان نیاز به داروها در ریکاوری، بدو ورود به بخش و ساعتهای 6 و 12 و 18 و 24 بعد از عمل ثبت شد. یافته ها: این مطالعه نشان داد که درجه PONV و میزان نیاز به متوکلوپرامید در 24 ساعت اول بعد از عمل به ترتیب در فاز لوتئال و فولیکولار بیشترین و در فاز قاعدگی و قبل از قاعدگی کمترین میزان را دارد، (64/41 X2= ، 001/0 p< ) (9/32 X2= ، 001/0 P< ). همچنین درجه درد و میزان مپریدین مورد نیاز جهت کنترل آن در 24 ساعت اول در فاز لوتئال و فولیکولار بیشترین و در فاز menstrual و Premenstrual کمترین میزان است (به ترتیب 001/0 P< ، 2/22 X2= ، 001/0 P< ، 6/41 X2= ). Ø بحث و نتیجه گیری: این مطالعه نشان داد که انجام کلیه عمل های جراحی بر اساس فاز سیکل قاعدگی ممکن است بروز PONV و دردمندی و میزان تجویز داروهای بعد از عمل و هزینه های بیمارستانی را کاهش دهد.

Title: وضعیت حفاظت و ایمنی پرسنل آزمایشگاههای تشخیص طبی در رابطه با ید رادیواکتیو

http://yafte.lums.ac.ir/article-1-22-fa.html

مقدمه: ید 125 از جمله مواد رادیو اکتیو است که در آزمایشگاههای تشخیص طبی در روش رادیو- ایمونواسی (RIA) برای سنجش های هورمونی مورد استفاده قرار می گیرد. در بسیاری از آزمایشگاههاتعدادی از شاغلین با این ماده در تماس هستند. در مواجهه با این ماده خصوصاً مواجهه داخلی ، نگرانی از عوارض تشعشعات یونیزان وجود دارد . بنابراین اقدام های حفاظتی در برابر چنین مخاطراتی الزامی است. با توجه به اینکه تاکنون در کشور هیچ بررسی در مورد مواجهه با ید 125 انجام نشده این مطالعه طراحی و اجرا گردید. مطالعه حاضر سعی دارد با نگرشی مقطعی وضعیت رعایت استاندارد های حفاظتی در رابطه با ید 125 را در تعدادی از آزمایشگاههای تشخیص طبی بررسی نماید. مواد و روش ها: مطالعه در 13 آزمایشگاه در سطح شهر قزوین در پاییز سال 84 انجام شده و 51 نفر از پرسنل این آزمایشگاهها که در محیط کار با ید 125 سر و کار داشتند مورد بررسی قرار گرفتند. روش کار به صورت استفاده از چک لیست حفاظتی بود که توسط پژوهشگر در محل آزمایشگاه تکمیل شد. برای بررسی میزان رعایت تناسب سلامت و شغل از کلیه نمونه ها سابقه پزشکی،تستهای آزمایشگاهی مانند CBC و TSH ، سابقه خانوادگی سرطان به ویژه در بستگان درجه اول و دوم اخذ گردید، در نهایت از نمونه ها معاینه بالینی به عمل آمد . یافته ها: از نظر رعایت اقدامات حفاظتی مشاهده گردید که 82 % افراد از دستکش مناسب استفاده کرده و 61% از خوردن ، آشامیدن واستعمال دخانیات ومواد آرایشی در محیط آزمایشگاه پرهیز می کنند ، اقدام فوری در رفع آلودگی های سطوح کاری در 90% وشستشوی دست ها در 94% موارد اجرا می شد. افراد در حین کار در همه موارد با ویالهای باز سر و کار داشتند که امکان ایجاد آئروسل وجود داشت، فقط 2% افراد از سطوح جاذب بر روی میز کار استفاده می کردند و میزان استفاده از هود مناسب 54% بود. در هیچ موردی ، معاینات بدو استخدام (از نظر تناسب برای شکل) و معاینات ادواری برای حفظ سلامتی انجام نگرفته بود. بحث و نتیجه گیری: وضعیت رعایت استانداردهای حفاظت فردی در آزمایشگاههای مورد مطالعه مناسبتر از اقدامات حفاظتی مدیریتی بود، انتظار می رود باآموزش کارکنان و مدیران و همچنین جدیت در بازرسی از آزمایشگاهها بتوان شرایط بهتری را به وجود آورد .

Title: غربالگری اختلال افسردگی در جمعیت 64 ـ 20 ساله شهر خرم‌آباد

http://yafte.lums.ac.ir/article-1-23-fa.html

مقدمه: افسردگی یکی از شایع‌ترین اختلالات روانپزشکی و از علل شایع ناتوانی و مرگ زودرس است. این اختلال همچنین باعث افزایش هزینه‌های اقتصادی تحمیل شده به فرد و جامعه می‌گردد. اختلال‌های روانی عامل پانزده درصد ناتوانی‌ها و مرگ‌های زودرس در کشورهای رو به رشد هستند. بر اساس گزارش مشترک WHO و بانک جهانی، اختلال افسردگی در صدر ده علت مهم ناتوانی و از کار افتادگی در جهان است. بنابر این شناسایی این اختلال در افراد و برخورد صحیح و درمان به موقع افسردگی آنان برای فرد، خانواده و جامعه بسیار ارزشمند است. مواد و روش ها: این پژوهش یک مطالعه توصیفی مقطعی با هدف غربالگری اختلال افسردگی در جمعیت 64 ـ 20 ساله شهر خرم آباد بوده است. جامعه آماری پژوهش شامل کلیه ساکنین شهری در مناطق دوگانه شهرداری خرم‌آباد بود. ابزار گردآوری اطلاعات پرسشنامه استاندارد شده بک با 21 پرسش بوده است که اختلال افسردگی را می‌سنجد. جمعیت هر یک از نواحی سه‌گانه در مناطق 2 و 1 شهرداری بصورت خوشه‌ای و چند مرحله‌ای انتخاب شدند. روش نمونه‌گیری مناطق خوشه‌ای و روش نمونه‌گیری خانوار نمونه‌گیری سیستماتیک بوده است. اطلاعات جمع‌آوری شده با روش آماری مجذور کای مورد تجزیه و تحلیل قرار گرفت. یافته ها: از مجموع 2740 نفر 4/33% دارای علائم اختلال افسردگی بودند که از این میان 25% دارای افسردگی خفیف، 1% شدید و 4/7% متوسط بودند. بیشترین میزان افسردگی بین 65 ـ 50 سالگی و کمترین میزان 46 ـ 30 سالگی بود. افسردگی در زنان با اختلاف ناچیزی بیشتر از مردان بود (3/34% در مقابل 4/32%). بیشترین میزان افسردگی در افراد بیوه 3/43% و کمترین آن در متأهلین 29% بود. بیشترین میزان افسردگی در مشاغل مختلف، در کارگران ساده 6/47% و کارگران تخصصی 2/43% و کمترین آن در کارمندان حرفه‌ای با 2/16% بوده است. بر حسب تحصیلات بیشترین افسردگی با 4/17% در مقاطع سیکل و دیپلم و کمترین آن به ترتیب در افراد بی‌سواد و یا با تحصیلات کم 1/7% و 6/8% با تحصیلات دانشگاهی بود. کمترین میزان افسردگی با 5/3% در افراد با درآمد بالای 200 هزار تومان و بیشترین میزان افسردگی با 7/29% در افراد با درآمد کمتر از 200 هزار تومان بوده است. همچنین بین سن، تأهل، میزان تحصیلات، شغل، میزان درآمد خانوار، نوع مسکن و افسردگی ارتباط معنی داری بدست آمد (001/0 p< ) ولی بین جنس، سکونت در مناطق مختلف و افسردگی ارتباط معنی داری وجود نداشت. بحث و نتیجه گیری: ارائه خدمات بهداشت روانی مسئله‌ای است که در کشورها کمتر به آن توجه شده است. اما تجارب پزشکان و مطالعات همه‌گیرشناسی نشان می‌دهد که اختلال‌های روانی از بسیاری جهات بخصوص از نظر فراوانی و شیوع و همچنین از لحاظ نیروهای انسانی کمتر از بیماریهای عفونی نیستند. بنابر این شناسایی این افراد، برخورد صحیح و درمان به موقع افسردگی آنان برای افراد، خانواده و جامعه بسیار ارزشمند می‌باشد و منجر به کاهش زیان‌های مادی و معنوی ناشی از آن می‌شود.

Title: بررسی ویژگیهای شخصیتی و روانشناختی زنان و دختران فراری نگهداری شده در مراکز مداخله در بحران های اجتماعی استان لرستان

http://yafte.lums.ac.ir/article-1-24-fa.html

مقدمه : محیط خانواده اولین محلی است که کودک استعدادها و توانائیهای خود را در آن محیط شناسائی نموده و آنها را رشد و پرورش میدهد. چنانچه خانواده نتواند محیط مناسبی را برای رشد و تکامل شخصیت کودک خود محیا نماید، اساس کجروی های اجتماعی را در او پایه گذاری می کند. خانواده به عنوان یکی از عوامل اجتماعی و شخصیت به عنوان یکی از عوامل درونی مطرح، می توانند، زمینه مناسبی را برای رفتارهای بزهکارانه ایجاد نمایند، لذا انجام پژوهش حاضر با هدف بررسی ویژگیهای شخصیتی و روانشناختی زنان و دختران فراری نگهداری شده در مراکز مداخله در بحران های اجتماعی استان لرستان در سال 1382 انجام گرفت. مواد و روشها: مطالعه حاضر یک مطالعه توصیفی، تحلیلی است. نمونه گیری به روش سرشماری بوده و کلیه دختران و زنان نگهداری شده در مراکز مداخله در بحران در طول یک سال مورد بررسی قرار گرفتند. جهت جمع آوری داده ها در این تحقیق از یک پرسشنامه دو قسمتی شامل مشخصات دموگرافیک و عوامل خانوادگی و پرسشنامه استاندارد شده SCL 90 استفاده شد. کلیه پرسشنامه ها از طریق مصاحبه بالینی توسط روانشناس بالینی تکمیل گردیدند. اطلاعات پس از جمع آوری با استفاده از نرم افزار SPSS مورد ارزیابی قرار گرفت. یافته ها : از 73 نمونه ای که در این تحقیق مورد بررسی قرار گرفتند اکثریت در رده سنی زیر 20 سال، خانه دار و دارای تحصیلات ابتدائی و راهنمایی بودند، اکثریت از فرزندان وسط خانواده بوده و سابقه مهاجرت از روستا به شهر داشتند. برخی از واحدهای مورد پژوهش سابقه زندان، وابستگی به مواد مخدر و انجام اعمال خلاف قبلی داشتند. برخی از اکثریت متأهلین علت فرار خود را ازدواج اجباری و مجردها درگیری شدید و کشمکش با والدین را علت اصلی فرار خود ذکر نمودند. همچنین یافته های تحقیق نشان داد 3/46% از واحدهای مورد پژوهش دارای فوبیا (ترس مرضی)، 3/64% دارای اختلال سایکوتیزم (روانپریشی)، 5/83% دارای اختلال هیپوکندریازیس (خود بیمار پنداری)، 4/90% دارای اختلال وسواسی جبری، 2/82% دارای افسردگی، 9/84% دارای اضطراب بیمارگونه، 6/68% پرخاشگری، 41% دارای اختلال پارانوئیدی و 2/56% دارای مشکل حمایت در روابط متقابل بودند. نتایج مطالعه نشان داد که بین فرار دختران و زنان با متغیرهایی مثل درگیری و کشمکش با والدین، ازدواج اجباری، بیسوادی یا سطح سواد پایین والدین، بیکاری و نداشتن درآمد والدین و سطح اقتصادی پایین خانواه ارتباط معنی دار وجود دارد. همچنین بین ویژگیهای شخصیتی و عاطفی با فرار ارتباط معنی داری بدست آمد. Ø بحث و نتیجه گیری : خانواده در بروز بزهکاری نوجوان نقش اساسی و حیاتی دارد، لذا آموزش به خانواده ها جهت برقراری روابط گرم و صمیمی با بچه ها و توجه به خواسته ها و نیازهای روانی آنها توصیه می شود.

Title: رابطه همی آنستزی، همی نگلکت و همی آنوپسی با مرتبه ناتوانی

http://yafte.lums.ac.ir/article-1-25-fa.html

مقدمه: یافته های بالینی تاثیر گذار بر ناتوانی بیماران سکته مغزی حائز اهمیت است. زیرا وجود این عوامل می توانند در پیش آگهی و بهبودی بعدی بیمار تاثیر گذار باشند. مواد و روشها: بیماران پی در پی مبتلا به آنفارکت مغزی شده و بیمارانی که به طور متعدد در بیمارستان ولیعصرخراسان بستری شده بودند در سال 1384 در این مطالعه قرار گرفتند. وجود همی هیپوستزی، همی آنستزی، همی نگلکت و همونیموس همی آنوپسی در بیماران بررسی شد. مرتبه ناتوانی بیمار 72 ساعت بعد از سکته بر اساس معیار رانکین تعیین شد. آزمون T و فیشر برای تحلیل آماری میانگین مرتبه ناتوانی بکار رفت و p کمتر از05/0 معنی دار تلقی شد. یافته ها : 329 بیمار با سکته مغزی بررسی شدند. در گروه مورد مطالعه4/37% همی هیپوستزی، 8/13% همی آنستزی، 9/7% همی نگلکت و 3/7% همونیموس همی آنوپسی داشتند. میانگین ناتوانی در بیماران با همی آنستزی بطور معنی داری بیشتر از سایر بیماران بود (001/0 (p< . میانگین ناتوانی در افراد با همی هیپوستزی، همی نگلکت و همونیموس همی آنوپسی نسبت به افراد بدون آن تفاوت معنی داری نداشت و بترتیب 44/0 p= ، 23/0 p= و 83/0 p= بود. میانگین ناتوانی در بیماران با تریاد همی آنستزی، همی نگلکت و همونیموس همی آنوپسی بطور معنی داری بیشتر از سایرین بود (001/0 p< ). بحث و نتیجه گیری : همی آنستزی موثرترین یافته بالینی تاثیر گذار بر ناتوانی مبتلایان به سکته مغزی است. وجود تریاد بالینی فوق تشدید کننده میانگین ناتوانی در این بیماران است.

Title: مقایسه اثر برونکودیلاتوری آلبوترول از طریق اسپیسر و نبولایزر، در درمان بیماران مبتلا به حمله آسم

http://yafte.lums.ac.ir/article-1-26-fa.html

مقدمه : آسم یک بیماری التهابی مزمن مجاری هوایی است که با افزایش پاسخ‌دهی مجاری تراکئوبرونکیال، مشخص می‌شود. درمان انتخابی حمله حاد آسم، استفاده از آگونیست‌های استنشاقی کوتاه اثر مانند آلبوترول است که از طریق نبولایزر یا محفظه آسم یار( اسپیسر )مصرف می‌شود. دو مطالعه حاضر با هدف بررسی مقایسه ای اثرات درمانی آلبوترول با استفاده از نبولایزر و محفظه آسم یار در بیماران مبتلا به حمله حاد آسم مراجعه کننده به اورژانس بیمارستان مباشر کاشانی همدان انجام شد . مواد و روش‌ها: این مطالعه بصورت کارآزمایی بالینی از نوع قبل و بعد، بر روی 60 بیمار مبتلا به حمله حاد آسم انجام شد. معیارهای ورود به مطالعه، سن 15 تا 65 سال، همکاری جهت انجام مانور اسپیرومتری توسط بیمار وحجم گاز خارج شده طی اولین ثانیه بازدم کمتر از 65 درصد قابل پیش‌بینی دراسپیرومتری بود. معیارهای خروج از مطالعه نیز ابتلا به COPD ، بیماری قلبی- عروقی، پنومونی، پنوموتوراکس و یا سایر بیماری‌های سیستمیک و ریوی غیر از آسم برونشیال، نارسایی شدید تنفسی همراه با سیانوز و یا کنفوزیون، خانم‌های باردار و مصرف کنندگان دخانیات بود. بیماران به طور تصادفی به دو گروه تقسیم شده که بیماران هر دو گروه، به مقدار چهار دوز آلبوترول دریافت کردندکه دوز اول بلافاصله پس از انجام اسپیرومتری و سپس به فواصل نیم ساعته در طی دو ساعت اول بود. FEV1 ، FVC و % MEF25-75 هر دو گروه در بدو مراجعه و سپس 15 دقیقه پس از تجویز هر دوز دارو، محاسبه شد (1، 2). یافته‌ها: آنالیز واریانس فاکتوریال مقایسه اثربخشی برونکودیلاتوری آلبوترول با دو روش استفاده از نبولایزر و اسپیسر نشان داد که هر دو روش استفاده از آلبوترول در تغییرات FEV1 ، FVC ، FEV1/FVC و MEF25-75 بیماران مبتلا به حمله حاد آسم اثر یکسان دارند (05/0 P> ). تغییرات FEV1 ، FVC ، MEF25-75 و FEV1/FVC درنوبت‌های درمان، متفاوت بوده (05/0 P< ) و تفاوتی بین اثردرمانی دوروش استفاده ازآلبوترول،درنوبت‌های مختلف درمان وجود نداشت (05/0 P> ). بحث و نتیجه‌گیری: جهت درمان بیماران مبتلا به حمله حاد آسم، یک آگونیست انتخابی مانند آلبوترول، می‌تواند به هرکدام از دو روش نبولایزر و یا اسپیسر مورد استفاده قرار گرفته و اثرات برونکودیلاتوری یکسانی، را ایجادنماید. با در نظر گرفتن قیمت ارزانتر شکل اسپری دارو در مقایسه با شکل محلول آن و از طرفی در دسترس نبودن دستگاه نبولایزر در برخی از مراکز درمانی،استفاده از شکل اسپری آلبوترول با محفظه آسم یار بر شکل نبولایزر آن ارجح می باشد.

Title: مقایسه میزان شکست آیودی های CU safe 300 و CU t 380 A طی سالهای 84-1380

http://yafte.lums.ac.ir/article-1-27-fa.html

مقدمه: آیودی یکی از روشهای مفید ومؤثرپیشگیری از بارداری است.امروزه انواع مختلف آیودی در بازار موجود می باشد که رایج ترین نوع مورد مصرف در مراکز بهداشتی درمانی کشورمان نوع CU t 380A می باشد. براساس مطالعات انجام شده انواع جدیدتر آیودی از جمله نوع CU safe 300 مؤثرتر وکم عارضه تراز انواع قبلی است وبه همین دلیل بسیار مورد پذیرش متقاضیان می باشد. مطالعه ای که توسط پژوهشگر وهمکاران چهارسال پیش در این زمینه انجام شد نشان داد عوارض جانبی زودرس آیودی از جمله خونریزی، درد و دفع خود به خودی آیودی طی چهار ماه اول بعد از جایگذاری در انواع CU safe 300 بسیار کمتر از انواع CU t 380 A بود. مطالعه حاضر جهت مقایسه عوارض دیررس این دو نوع آیودی بعمل آمد شاید نتایج آن بتواند راهگشایی برای گسترش استفاده از این روش مفید تنظیم خانواده گردد. مواد و روش ها: این بررسی که یک مطالعه همگروهی است در آن 246 نفر از زنانی که طی چهارسال بصورت دو گروه (123 نفری) از آیودی CU safe 300 و CU t 380A استفاده نموده اند مورد بررسی مجدد قرار گرفتند. با استفاده از پرسشنامه وطی مصاحبه ای حضوری در مورد عوارض دیررس از جمله دفع خود بخودی و حاملگی ناخواسته، اطلاعات جمع آوری شد. در نهایت اطلاعات با استفاده از نرم افزار SPSS وآزمون مجذور کای وتی زوج مورد تجزیه وتحلیل قرار گرفت. یافته ها: نتایج مطالعه نشان داد آیودی CU safe 300 بسیار مؤثر تر وکم عارضه تر از انواع CU t 380A می باشد ومقایسه میزان شکست این دو آیودی با استفاده از آزمون تی زوج تفاوت معنی داری نشان داد. بحث و نتیجه گیری: با توجه به نتایج بدست آمده از این پژوهش وهمخوانی با سایر مطالعات آیودی جدید CU safe 300 بسیار مؤثرتر وکم عارضه تر از آیودی های رایج است وتوصیه می شود در صورت امکان جایگزین آیودی های قدیمی تر گردد ویا در کنار سایرانواع مورد استفاده قرار گیرد

Title: ریسک ناشی ازتابش های یونساز در دوران بارداری

http://yafte.lums.ac.ir/article-1-28-fa.html

کلیات موضوع: کشف اشعه ایکس توسط رونتگن در نوامبر 1895 میلادی و بدنبال آن نتایج سودمندی که از انجام آزمایشات رادیولوژی تشخیصی، درتشخیص نهایی بیماریها حاصل شد، استفاده روزافزون از اشعه ایکس را بدنبال داشت. از آنجاییکه تابش گیری های پزشکی عمده ترین بخش تابش گیری های مصنوعی را شامل می شود بلافاصله چند ماه پس از کشف اشعه ایکس، ناراحتی های چشمی و درماتیت شدید و پیشرونده در دست های پزشکان و متخصصین فیزیک ایجاد گردید، اما آثاردیررس ناشی از تابش گیری تقریباً 20 سال پس از کشف اشعه ایکس مشخص گردید. تاریخچه: برطبق بررسی های بعمل آمده تابش های یونساز خطر بالقوه ای در رشد جنین محسوب می گردند، لذا اجتناب از تابش گیری های غیرضروری در خانم های باردار به جز در مواردی که اندیکاسیون بالینی قوی وجودداشته باشد، توصیه می گردد، در این راستا رعایت استانداردهای رایج حفاظت در برابر اشعه شامل توجیه پذیری عمل، بهینه سازی روش های حفاظتی و محدودکردن دز اشعه دریافتی، با توجه به یکسان نبودن میزان حساسیت جنین درمراحل مختلف رشد و تکامل جهت جلوگیری از بروز آثار ناشی از تابش گیری الزامی است. بحث و نتیجه گیری: باتوجه به فوایدغیرقابل انکارپرتوهای ایکس در تشخیص و درمان بیماری ها، گسترش روزافزون بکارگیری این پرتوها را شاهد هستیم در عین حال باتوجه به آثار سوماتیکی و ژنتیکی این پرتوها، لزوم بهره گیری از آخرین روش های حفاظت پرتوی الزامی است. حفاظت دربرابر اشعه نه تنها یک موضوع علمی، بلکه یک بحث فلسفی، اخلاقی وعقلایی است، لذامی بایست جهت جلوگیری از آثار زیانبار تابش گیری در جنین، ازتابش گیری غیر ضروری خانم های باردار اجتناب نمود، مگر اینکه اندیکاسیون بالینی قوی داشته باشد.

Title: آپاندیسیت مزمن در بیماری با 15 سال درد شکمی

http://yafte.lums.ac.ir/article-1-29-fa.html

مقدمه: آپاندیسیت ‏حاد یک بیماری شناخته شده است که تمامی کادر درمانی به نوعی از آن آگاهی دارند اما آپاندیسیت ‏ مزمن حتی در نزد بسیاری از پزشکان ناشناخته و گاهی به سختی به آن باور دارند. اگر چه تعداد بیماران مبتلا به آپاندیسیت ‏ مزمن در مقابل مبتلایان به آپاندیسیت ‏ حاد بسیار کم است اما نباید به واسطه شیوع کم بیماری، از آن غافل بود. علائم بالینی این بیماران شامل دردهای شکمی مزمن، طولانی مدت وعود شونده که بیشتر در ناحیه ربع تحتانی وراست شکمی است ودر اقدامات پاراکلینیکی عمده بیماران (ادرار، مدفوع و سونوگرافی شکم ولگن) نکته پاتولوژیکی خاصی وجود ندارد. دراین گونه بیماران درصورت تشخیص آپاندیسیت ‏ مزمن وآپاندکتومی علائم بیماران برطرف وبیماران بهبودی می یابند. معرفی مورد: بیمار، مرد 57 ساله ایی بود که حدود 15 سال از درد شکمی مزمن درناحیه ربع تحتانی راست شکم شکایت داشت ودرتمامی اقدامات تشخیصی نکته پاتولوژیکی یافت نشده بود ولی باتشخیص آپاندیسیت ‏ مزمن تحت عمل جراحی آپاندکتومی قرارگرفت و بهبودی کامل حاصل نمود. بحث و نتیجه گیری: در بیمارانی که از درد شکمی طولانی و مزمن خصوصاً در ناحیه ربع تحتانی راست شکم شکایت دارند، باید به فکر آپاندیسیت مزمن بود.
